# MAGLIO study: epideMiological Analysis on invasive meninGococcaL disease in Italy: fOcus on hospitalization from 2015 to 2019

**DOI:** 10.1007/s11739-023-03377-7

**Published:** 2023-08-01

**Authors:** Carlo Tascini, Raffaella Iantomasi, Francesco Sbrana, Ciro Carrieri, Daniela D’Angela, Silvia Cocchio, Barbara Polistena, Federico Spandonaro, Eva Agostina Montuori, Vincenzo Baldo

**Affiliations:** 1 U.O. Malattie Infettive, Dipartimento di Medicina dell’Università di Udine, Università di Udine e Azienda Sanitaria Universitaria Integrata di Udine, Via Pozzuolo, 330, 33100 Udine, Italy; 2 Vaccine Medical Department, Pfizer, Rome, Italy; 3 U.O. Lipoapheresis and Center for Inherited Dyslipidemias, Fondazione Toscana Gabriele Monasterio, Pisa, Italy; 4 C.R.E.A. Sanità e Università di Roma Tor Vergata, Rome, Italy; 5 Department of Cardiac Thoracic Vascular Sciences and Public Health, Public Health Section, University of Padua, Via Leonardo Loredan 18, 35131 Padua, Italy; 6 Università Telematica San Raffaele di Roma e C.R.E.A. Sanità, Rome, Italy

**Keywords:** Invasive meningococcal disease, Italy, Hospitalization, Mortality, Costs

## Abstract

This study analyzed hospital admissions for invasive meningococcal disease (IMD) in epidemiological and economic terms in Italy from 2015 to 2019. The volume of acute admissions for meningococcal diagnosis was analyzed in the period from 2015 to 2019. IMD admissions were identified by ICD-9-CM diagnoses. Costs were assessed using current DRG tariffs. In 2019, a total of 237 admissions for meningococcal disease were recorded in Italy. The mean age of patients was 36.1 years. Lumbar puncture was reported in only 14% of hospital discharge forms. From 2015 to 2019, there was a mean annual reduction of − 1.2% nationally for IMD hospitalizations. For 2019, the total costs for acute inpatient admissions were €2,001,093. Considering annual incidence due to IMD, a significant decrease was noted in the age group from 0 to 1 year (*p* = 0.010) during 2015–2019. For all years, mortality associated with meningeal syndrome was lower compared to septic shock with or without meningitis. From 2015 to 2019, hospitalizations for IMD appear to be decreasing slightly in Italy, even if mortality remains high. Favorable trends in hospitalizations for IMD were seen in the 0–1-year age group, which may be attributable to increased vaccination. Costs of hospitalizations for IMD remain high.

**Supplementary Information:**

The online version contains supplementary material available at 10.1007/s11739-023-03377-7.

## Introduction

Invasive meningococcal disease (IMD) is a life-threatening illness caused by the pathogen *Neisseria meningitidis* [1]. IMD affects all ages, can progress rapidly, and have several manifestations; the most common are meningitis, septicemia, or their combination [1]. The most ominous manifestation is septic shock with purpura fulminans that depends on the causative strain [1]. Survivors may experience serious lifelong sequelae [2]. Of all *N. meningitidis* serogroups identified, five are the most often responsible for IMD (serogroups A, B, C, Y, and W), but the epidemiology of IMD varies around geographical areas and having a low, moderate, or high endemicity [3].

In Europe, based on the last Annual Epidemiological Report available from ECDC the notification rate of cases with confirmed IMD was 0.6 cases per 100,000 population, similar to the notification rate in previous years but a slight increase compared to 2014 (0.5 cases per 100,000) [4]. The distribution of the meningococcal serogroups varies considerably, both geographically and temporally, around the world. For example, Meningococcal serogroup B (MenB) dominates in many parts of the world, including Europe, while serogroup C became an important concern when it emerged rapidly in the late 1990s although its incidence decreased substantially following the introduction of a conjugated vaccine in immunization programs in many European countries [5]. Isolation of serogroups Y and W has historically been lower in Europe, compared to B and C, although an hypervirulent MenW strain has been responsible for increases in IMD cases in various European countries [4, 6–8]. Recent evidence indicates that meningococcal serogroup Y (MenY) has continued to increase in northern Europe and the proportion of IMD attributable to MenY remains high in Scandinavian countries even if MenW is the second cause of IMD in Europe [4, 9, 10]. The incidence of IMD is highest in infants, followed by young children; however, a secondary peak may occur among adolescents and young adults [11].

In Italy, MenB is the most common serogroup, The MenB serogroup showing an increasing trend during recent years, from the 36.3% in 2016 to 67% of all cases in 2020 [12, 13]. Of note, in the two major at-risk age groups, serogroup B represented 81% of cases in those 0–4 years of age and 87.5% among young adults (15–24 years of age) [14]. Overall, an analysis of consolidated data shows that the incidence of IMD has remained stable over the past decade [12, 15]. Vaccination programs constitute an important factor affecting IMD epidemiology including serogroup prevalence. In Italy, the current recommendations from the National Immunization Plan 2017–2019 recommend a MenB vaccination for infants and MenC/MenACWY, depending on the region, at 13–15 months of age [16–22].

An unexpected outbreak of MenC in young adults occurred in 2015 due to a cluster in Tuscany, which recurred in the early months of 2016 [23]. More recently, two other outbreaks occurred: five cases of MenB in Sardinia in 2018 [24] and the most recent, between December 2019 and January 2020, with six cases of IMD infected with MenC clonal complex cc11 identified in a limited area in the northern part of Italy [25]. A reactive vaccination control strategy with a MenB vaccine in Sardinia, and a quadrivalent conjugate vaccine against serogroups A, C, W, and Y (MenACWY) in north Italy were implemented, vaccinating the outbreak area population [6].

While outbreaks are unpredictable by definition, preventive strategies for IMD require a detailed understanding of the current incidence of IMD and serogroup distribution. This information is provided by surveillance systems, although the real burden of IMD, comprehensive of clinical outcomes and sequalae, disease management costs (both direct and indirect), and social impact are rarely described. These elements are key for effective prophylactic strategies and targeted control interventions to defeat IMD [26]. The aim of this study is to analyze hospitalizations for IMD in Italy for the period 2015–2019 in epidemiological, clinical, and economic terms.

## Materials and methods

### Setting and data source

We conducted this retrospective study in Italy, which has a population of 59.6 million. We analyzed the acute ordinary hospital discharge records (HDRs) of public and accredited private hospitals from 2015 to 2019. HDRs include the following data: age, gender, place of residence, type of the hospital, date of admission, surgical and other procedures, and date and type of discharge (at home, at rehabilitation facilities, death, etc.). Each HDR contained one primary diagnosis (or first-listed diagnosis concerns the main condition identified during the patient's hospital stay) and up to five secondary diagnoses based on the diagnostic codes of the International Classification of Diseases, Ninth Revision, Clinical Modification (ICD-9-CM).

### Acute admissions for IMD

The volume of acute admissions for meningococcal diagnosis is analyzed in terms of number of admissions and related dynamics recorded in the period 2015–2019. The standardized admission rate, the related trend at the geographical divisions level, and the incidence of admissions by age and sex group are evaluated. Hospital admissions for IMD were identified by the ICD-9-CM diagnoses (main or secondary) reported in Supplementary Table 1.

Based on adjunctive ICD-9-CM diagnosis such as septic shock, sepsis, for the years 2015 to 2019, all cases identified with the ICD-9-CM listed in Supplementary Table 1 were grouped in two main categories: (i) meningococcal meningitis/encephalitis only and (ii) meningococcemia or septic shock with or without meningitis. The first category includes all admissions related to ICD-9-CM diagnosis codes 036.0 and 036.1 (main and secondaries), the second one all admissions associated to diagnosis 036.2, 036.3, and all diagnoses of sepsis or septic shock (ICD-9-CM 771.81, 785.52, 785.59, 995.91, 995.92).

### Hospitalization rate and length of stay

Based on the total number of hospital admissions concerning Italian residents in each year considered, annual hospitalization rates were calculated by dividing the annual number of hospitalizations by the population in the year considered, according to the Italian statistical office, and expressing the rates as hospitalizations per 100,000 population. The length of hospital stays was calculated as the days elapsing between the dates of admission and discharge, and the mean hospital stay was calculated. The case fatality rate (CFR) was calculated by dividing the number of in-hospital deaths by the number of patients hospitalized with a diagnosis of IMD, expressed as a percentage.

### Estimated costs

The estimated cost to the health care system of hospital admission for IMD was calculated using the diagnosis-related groups (DRGs) of hospitalized patients. The DRGs depend on the patients' ICD classification at the time of their discharge from hospital, their age and gender, and the consumption of resources during their hospital stay. According to the DRG-based reimbursement system, every hospitalized patient belongs to a group of diagnostically homogeneous cases. Patients within each category are therefore similar in clinical terms and are expected to require the same level of hospital resources. As a result, patients in the same DRG are assigned the same reimbursement charges. All hospital stays were analyzed considering, for the same patients, only the first hospital admission. Any case of secondary hospitalization, transfer to other acute care institutions, and admission to rehabilitation institutions, associated with the same patient, was removed from the initial dataset for the years 2015 to 2019.

### Statistical analysis

Significant trends over the period considered were assessed as average annual percent changes (AAPC), a summary measure of the trend over a given fixed interval [27]. An AAPC of zero coincides with the hypothesis of a trend that is neither increasing nor decreasing. The 95% confidence interval (95% CI) was calculated and a *p* value < 0.05 was considered significant.

## Results

### Hospitalizations for IMD in 2019

In 2019, in Italy, a total of 237 admissions for meningococcal disease were recorded; of these, 93.7% were attributed to acute disease, while the remaining 6.3% were related to rehabilitation or long-term care facilities (Table 1). Considering hospitalizations, there were 215 (96.8%) acute inpatient admissions and 7 (3.2%) outpatient admissions. The mean age of patients was 36.1 ± 26.3SD years. Overall, acute admissions for IMD represents 0.004% of total acute inpatient admissions. Approximately two-thirds (71.1%) of acute inpatient admissions were associated with a main diagnosis of IMD and were related to two types of diagnoses: meningococcal meningitis (ICD-9-CM 036.0) and meningococcemia (ICD-9-CM 036.2). Other ICD-9-CM codes were less commonly found (Supplementary Table 2). Overall, 87.9% of cases were admitted to only four types of wards: infectious disease unit (49.3%), intensive care unit (17.7%), pediatric care unit (13.0%), and medical ward (7.9%). Lumbar puncture was reported in only 14% of hospital discharge forms, and 12.1% of forms did not report the procedure used for diagnosis of meningococcal disease.

**Table 1:** Characteristics of hospital admissions for IMD in Italy in 2019

Total admissions (*N*)	237
Acute disease (*N*, %)	222, 93.7%
Rehabilitation/long-term care facilities (*N*, %)	15, 6.3%
Acute inpatient admissions (*N*, %)	215, 96.8%
Outpatient admissions (*N*, %)	7, 3.2%
Mean age (years)	36.1 ± 26.3 DS
Diagnosis	
Main diagnosis of IMD (*N*, %)	153, 71.1%
Meningococcal meningitis	114, 74.5%
Meningococcemia	39, 25.5%
Admission ward	
Infectious disease unit	106, 49.3%
Intensive care unit	38, 17.7%
Pediatric care unit	28, 13.0%
Medical ward	17, 7.9%

The national acute ordinary inpatient rate was 0.35 admissions per 100,000 inhabitants, with the highest of 0.42 cases per 100,000 inhabitants in the northwest; the center with 0.39 per 100,000 inhabitants, the northeast with 0.33, and the south with 0.29. The inpatient rate was also stratified by age group (Supplementary Fig. 1). The highest rate was found in infants below one year of age (3.48 per 100,000) followed by 1–4 years (1.05 per 100,000) and 15–19 years (0.84 per 100,000). Moreover, the acute inpatient rate by gender was 0.40 admissions per 100,000 males and 0.30 admissions per 100,000 females. The mean duration of hospitalization was 13.7 ± 12.3 SD days with significant variation among the Italian regions (3.0–19.3 days). The group over 65 years of age had the longest mean hospitalization (19.1 days), while the lowest was 8.1 days in those 20–24 years old (Table 2). Analysis of the discharge type revealed that most were “discharged at home” (64.7%), followed by "transfer to another acute care institution" in 14.4% of cases and "patient death" in 11.6% of cases.

**Table 2:** Mean length of hospital stay by age group, sex, and clinical manifestation in 2019

	Mean length of hospital stay (days)
Age group (years)	
0	9.6 [1; 27]
1–4	8.6 [4,3; 12.5]
5–9	11.2 [4; 25.5]
10–14	8.8 [1; 11]
15–19	11.9 [3; 23]
20–24	8.1 [1; 22]
25–44	14.2 [9; 23]
45–64	15.3 [1; 58]
≥ 65	19.1 [8; 27.2]
Overall	13.7 [3; 19.3]
Male	13.3 [11.1–14.6]
Female	14.1 [10.2–16.6]
Meningococcal meningitis/encephalitis only	15.7 [11.5–17.7]
Meningococcemia or septic shock with or without meningitis	14 [9.8–15.9]

### Acute inpatient admissions from 2015 vs. 2019

To analyze the changes over 5 years in IMD-related admissions, hospital discharge forms were retrieved for the year 2015. Overall, 226 acute inpatient admissions were recorded in 2015, compared to 215 in 2019, for a mean annual reduction of − 1.2% nationally. The maximum reduction was observed in the northeast (− 8.5% mean annual change—m.a.c.) and the minimum in center (− 3.9% m.a.c.). In the northwest and south, there was an increase in acute inpatient admissions of 3.3% in both areas from 2015 to 2019. In the periods considered (2015 and 2019) there was a reduction of − 1.4% in the mean hospital stay for acute inpatient admissions (− 0.8 days), which was greater than the mean annual reduction recorded for all admissions (− 1.2%). In contrast, the mean age of inpatients increased from 2015 to 2019 by 5.2 years.

### DRG analysis

In 2019, the distribution of invasive meningococcal admissions was associated with a total of 26 DRGs; 77.7% of cases focus on 3 codes (Supplementary Table 3). The most commonly reported DRG was bacterial infections and tuberculosis of the central nervous system (54.4%), septicemia without mechanical ventilation > 96 h in age > 17 years (14.4%), and septicemia with age < 18 years (8.8%). At least one of these DRGs was present in 77.7% of cases. For the year 2019, the total costs for acute inpatient admissions were €2,001,093, calculated with the rates (inpatient) relating to all DRGs associated with hospital admissions listed in Supplementary Table 4. The above value was equal to 0.008% of the total value of all acute inpatient admissions. Furthermore, the mean cost of hospitalization was €9307.4 (+ 1.3% vs. 2015) with differences in the Italian regions ranging from a maximum of €12,442.7 in the center of the country to a minimum of €7429.1 in the northwest. Comparing the data to the number of residents, the per capita burden for acute admission was €31.6 per 1000 inhabitants, with a maximum of €44.1 in the center, followed by the northwest with €30.7, the northeast with €28.1, and the south with €27.1. When the costs were stratified by age group, the highest mean acute inpatient admission costs were seen in those over 65 years (€11,245) and the lowest in the 0–1 year range (€5997) (Supplementary Table 4).

### Trends in incidence and mortality from 2015 to 2019

**Fig. 1:**
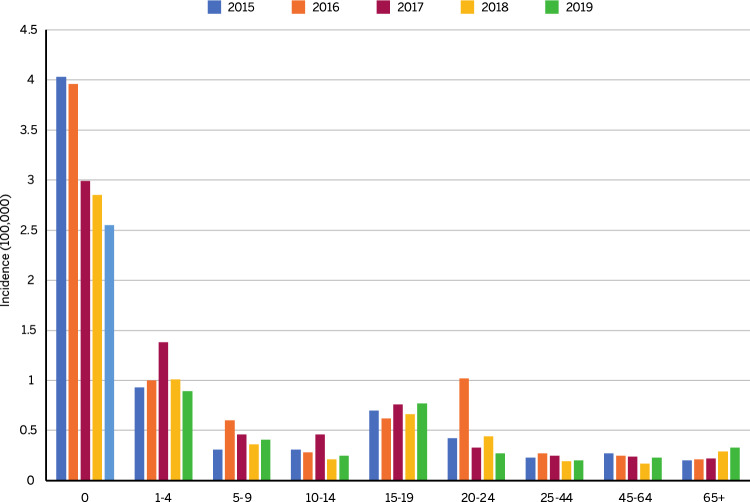
Incidence of admissions for IMD by age group from 2015 to 2019

**Fig. 2:**
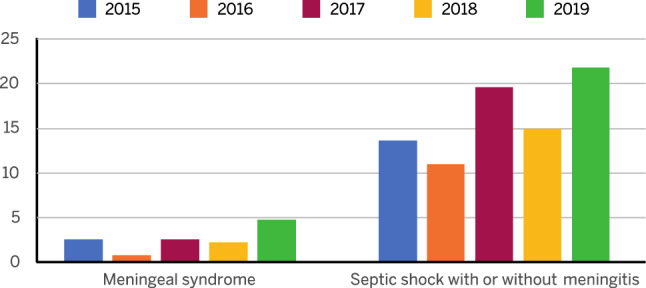
Percentage of hospitalized patients who died from “meningeal syndrome” or “septic shock” with or without meningitis from 2015 to 2019

For further analyses, all cases of secondary hospitalization, transfer to other acute care institutions, and admission to rehabilitation institutions, associated with the same patient, were removed from the dataset for the years 2015–2019. Cases admitted in December were only counted in the year of admission. In 2019, compared to 237 hospital admissions, 192 cases were found. Of these, 105 (54.7%) cases were in the macro-category of "meningeal syndrome," while the remaining 87 (45.3%) cases were related to "septic shock with or without meningitis." To evaluate the 5-year trend, the same analysis was done on the years 2018 (180 cases), 2017 (212 cases), 2016 (229 cases), and 2015 (201 cases). Annual incidences by age group from 2015 to 2019 are shown in Fig. 1. Of note, a statistically significant decrease was noted in the age group from 0to 1 year (AAPC: − 11.7; 95% CI − 17.4 to − 5.6; *p* = 0.01), while an increase was noted in those ≥ 65 years (AAPC: 14.2; 95% CI 5.5 to 23.5; *p* = 0.013). Differences were not apparent in any of the other age groups. For all years analyzed, mortality associated with meningeal syndrome was lower compared to septic shock with or without meningitis (Fig. 2). Moreover, for the period 2015–2019 the highest mean mortality was found for the age group ≥ 65 years (15.8%), and the lowest in those 1–4 years of age (6.7%) (Table 3). In 2019, the highest rate of mortality was seen in the 20–24 year age group (37.5%).

**Table 3:** Mean mortality rates in 2015–2019 by age group

Age group (years)	Mean mortality (%)					
	2015–2019	2015	2016	2017	2018	2019
0	6.7 (5)	10.0 (2)	0.0 (0)	6.7 (1)	8.3 (1)	10 (1)
1–4	4.3 (4)	6.3 (1)	5.3 (1)	8.7 (2)	0.0 (0)	0 (0)
5–9	5.3 (3)	0.0 (0)	5.9 (1)	7.7 (1)	12.5 (1)	0 (0)
10–14	5.1 (2)	0.0 (0)	0.0 (0)	8.3 (1)	0.0 (0)	20.0 (1)
15–19	5.9 (6)	5.0 (1)	0.0 (0)	4.5 (1)	10.5 (2)	9.1 (2)
20–24	7.8 (6)	0.0 (0)	6.9 (2)	0.0 (0)	7.7 (1)	37.5 (3)
25–44	8.9 (15)	14.7 (5)	2.4 (1)	8.3 (3)	7.1 (2)	14.3 (4)
45–64	10.4 (20)	7.0 (3)	5.1 (2)	17.5 (7)	9.7 (3)	12.8 (5)
≥ 65	17.4 (25)	9.5 (2)	22.7 (5)	17.9 (5)	13.5 (5)	22.2 (8)

During 5-year period, the number of cases of IMD reported by the National Health Service differed substantially and was consistently lower than those from hospital discharge forms (Table 4).

**Table 4:** Cases of IMD reported in hospital discharge forms and those reported by the National Health Service

Year	Cases reported in HDF (*n*)	Cases reported by NHS (*n*)	Change (%)
2015	201	187	− 7.0
2016	229	228	− 0.5
2017	212	197	− 7.1
2018	180	170	− 5.5
2019	192	190	− 1.0

## Discussion

Based on HDRs, the present retrospective study analyzed hospitalizations for IMD in Italy for the period 2015–2019. Overall, in 2019, there were 237 admissions with main or secondary invasive meningococcal diagnosis (0.36 per 100,000 inhabitants) with the vast majority being attributed to acute disease. Admission for IMD is mainly concentrated in the first year of life, followed by those aged 1–4 and 15–19 years. The presence of a second peak of incidence is common in several countries, with the rate in adolescent/young adults being generally 2 times or more of the rate in the overall population (among all age groups, including children) [28]. The difference in admission rates by macro-area (northwest, northeast, center, and the south) is in line with the notification rate interregional variability, noted by the Italian National Health Service. The North–South gradient could be linked to differences in the susceptibility and vulnerability of the population, to transmission dynamics, or to underdiagnosis/under-notification phenomena [12]. Nearly half of the cases were admitted to an infectious disease unit. We also analyzed changes in hospitalizations for IMD from 2015 to 2019. Over this period, there was a slight reduction in hospitalizations for IMD, with a mean annual reduction of 1.2%, and some differences in the different geographic regions can be noted.

In-hospital mortality occurred in roughly 12% of cases. Considering mortality from IMD during 2015–2019, it is notable that mortality associated with meningeal syndrome was lower than that due to septic shock with or without meningitis. This is in line with a previous study reporting that in patients with IMD mortality is higher from septic shock compared to mild meningococcemia or meningitis [29]. In other studies, mortality from IMD has been related to age higher than 50 years old, seizures, shock, and meningococcemia without meningitis [30]. We also found differences in mortality in different age groups. Over the analyzed period, mortality in patients ≥ 65 years was consistently higher than that in other age groups with the exception of 2016 and 2019. In the latter year, mean mortality was 22.2% in patients over 65 years and 37.5% in those aged 20–24 years. Mortality in younger ages was highly variable from year to year. Higher mortality has also been related with the appearance of a highly virulent strain of *Neisseria meningitidis* Serogroup C that appeared in the Tuscany region in 2015/2016 where septic shock at presentation was reported in nearly half of the cases [31]. Moreover, in the 61 patients with IMD in Tuscany from 2015 to 2016 (mean age 26 years), 67.3% recovered; all patients had received timely and appropriate antibiotic therapy [32]. The appearance of such virulent strains helps explain differences in mortality in different age groups over the time period evaluated, such as that which is evident in the present analysis in 2016 in those 20–24 years of age.

Of note, lumbar puncture was reported in only 14% of hospital discharge forms, while a similar percentage did not report the diagnostic test used. While it is possible that such a low percentage may in part be due to underreporting, it is also possible that lumbar puncture is not being performed in many patients, even if there is no contraindication. A study from the UK reported that around half of children with a suspected infection of the central nervous system did not receive lumbar puncture [33]. In this type of patients, lumbar puncture plays a useful role in both the diagnosis and management of the disease [34]. Furthermore, no reason linked to reimbursement policy in Italy could justify this underreported data. The reasons for such a low percentage of patients without lumbar puncture warrant further investigation.

In economic terms, the total burner of acute meningococcal admissions was over €2 million, of which 98.1% for acute cases, 1.8% for rehabilitation, and 0.1% for long-term care. Moreover, the costs of inpatient admissions increased by 1.3% per year from 2015 to 2019. However, the costs of acute inpatient hospital admissions for IMD represents 0.008% of the overall value of all hospital admissions in Italy [35]. The mean value of an acute inpatient admission is €9307 with a maximum of €11,245 in patients over 65 years of age. Overall, high regional variability emerged in terms of recourse to IMD admission. The direct costs are similar to those found by Scholz et al. who evaluated costs of invasive meningococcal serogroup B disease in a cohort of 343 patients reconstructed from the database of the German National Institute of Public Health in the period 2001–2016 [36]. Of note, the direct costs associated with hospitalization found in this study are higher than the costs used to evaluate the cost-effectiveness of implementation of a free-of-charge anti-MenB vaccination program for adolescents in Italy [37, 38].

A steady, consistent reduction in hospitalizations for IMD was also clearly evident in infants 0–1 years of age, from about 4 per 100,000 to around 2.5 per 100,000. The incidence of IMD fluctuates geographically and temporally by unpredictable epidemiologic variations [39]. However, epidemiologic changes in *N. meningitidis* can also be impacted by vaccination. For example, the incidence of MenC has decreased in European countries that introduced the meningococcal C conjugate (MCC) vaccine into their NIPs, while no substantial change has been observed in MenC in countries that have not [10]. Disease reduction in Italy and other countries has been also reported following the introduction of the 4CMenB vaccine [40]. This would appear to reinforce the success of MenB vaccination in Italy introduced in 2014 [15, 41]. It also reinforces the need to achieve high vaccination rates through collaboration with vaccination centers and pediatricians/general practitioners [42] as well as the need for a preventive strategy targeting all at-risk age groups. In particular, the data that support prompt introduction of anti-meningococcal B vaccination even in adolescence relate to the fact that the majority of cases of invasive meningococcal disease between 10 and 17 years are currently attributable to this agent in Italy; cases of invasive meningococcal disease have the highest lethality in adolescence and are already present in Italy and in other countries [43]. Moreover, a diffuse vaccination campaign could reduce, as reported in infant population, the hospitalization. This aspect protects population from IMD sever complication and may contribute to the cost reduction due to hospitalization itself.

Lastly, it is worth noting that over the 5-year period of the study, the number of cases of IMD reported by the NHS differed and was consistently lower than those from HDFs. Since there is an obligation for the NHS to report all cases of IMD, the reasons for this apparent discrepancy merit further attention. However, it has very recently been noted that the surveillance system in Italy has been improving in terms of timeliness and sensitivity and that it performs well overall [44].

Among the limitations of the present study, the serotype of hospitalized patients was not collected, which would have provided important information on epidemiological trends over the years. In addition, as mentioned, many procedures are not reported in the diagnostic codes used to retrieve data, such as lumbar puncture as discussed above.

In summary, herein we report that from 2015 to 2019 hospitalizations for IMD appear to be decreasing slightly, although mortality remains high and largely due to septic shock. Distinct differences are apparent in hospitalizations for IMD during that time period, and favorable trends were seen in the 0–1-year age group, which may be attributable to increased vaccination. Costs of hospitalizations for IMD nonetheless remain high.

### Supplementary Information

Below is the link to the electronic supplementary material.

Supplementary file1 (DOCX 376 KB)

## Data Availability

Not applicable.
