# Supplementary material for: MAGLIO study: epideMiological Analysis on invasive meninGococcaL disease in Italy: fOcus on hospitalization from 2015 to 2019
Source: Intern Emerg Med. 2023 Aug 1;18(7):1961–9. doi: 10.1007/s11739-023-03377-7 (PMC10543787; doi:10.1007/s11739-023-03377-7)
Supplement: Supplementary file 1 — Supplementary file1 (DOCX 376 KB) [file 11739_2023_3377_MOESM1_ESM.docx]

**MAGLIO study: epideMiology Analysis on invasive meninGococcaL disease in Italy - fOcus on hospitalization from 2015 to 2019**

Supplementary Figure 1. Mean hospitalization rates by age group in 2019.

Supplementary Table 1**.** Invasive meningococcal disease ICD-9-CM diagnoses (main or secondary).

| **ICD-9-CM** | ***Diagnosis*** |
| --- | --- |
| 036.0 | Meningococcal meningitis |
| 036.1 | Meningococcal encephalitis |
| 036.2 | Meningococcemia |
| 036.3 | Waterhouse-Friderichsen syndrome, meningococcal |
| 036.40 | Meningococcal carditis, unspecified |
| 036.41 | Meningococcal pericarditis |
| 036.42 | Meningococcal endocarditis |
| 036.43 | Meningococcal myocarditis |
| 036.81 | Meningococcal optic neuritis |
| 036.82 | Meningococcal arthropathy |
| 036.89 | Other specified forms of meningococcal infections |
| 036.9 | Unspecified meningococcal infection |

Supplementary Table 2. Acute hospital discharges by main diagnosis, 2019

| **ICD-9-CM** | **Number of discharges** | **Main diagnosis** | **% of all discharges** |
| --- | --- | --- | --- |
| 036.0 | 114 | Meningococcal meningitis | 53.0 |
| 036.2 | 39 | Meningococcemia | 18.1 |
| 518.81 | 9 | Acute respiratory failure | 4.2 |
| 785.52 | 7 | Gram-negative endotoxic septic shock | 3.3 |
| 995.92 | 6 | Sepsis, with acute organ dysfunction/multiple organ dysfunction/severe | 2.8 |
| 036.1 | 5 | Meningococcal encephalitis | 2.3 |
| 036.3 | 2 | Waterhouse-Friderichsen syndrome, meningococcal | 0.9 |
| 038.49 | 2 | Other septicemia from gram-negative microorganisms | 0.9 |
| 320.1 | 2 | Pneumococcal meningitis | 0.9 |
| 995.91 | 2 | Sepsis | 0.9 |
| 009.1 | 1 | Colitis, enteritis and gastroenteritis of presumed infectious origin | 0.5 |

| **Supplementary Table 3. Acute hospital discharges by DRG code, 2019** | | | |
| --- | --- | --- | --- |
| **ICD-9-CM** | **Number of discharges** | **DRG description** | **% of all discharges** |
| 560 | 117 | Bacterial infections and tuberculosis of the nervous system | 54.4 |
| 576 | 31 | Septicemia without mechanical ventilation ≥ 96 hours, age> 17 years | 14.4 |
| 417 | 19 | Septicemia, age <18 years | 8.8 |
| 542 | 8 | Ventilated tracheostomy ≥ 96 hours or major diagnosis not related to face, mouth and neck without major surgery | 3.7 |
| 565 | 7 | Diagnosis related to the respiratory system with assisted breathing ≥ 96 hours | 3.3 |
| 575 | 6 | Ventilated septicemia ≥ 96 hours, age> 17 years | 2.8 |
| 001 | 3 | Craniotomy, age> 17 years with CC | 1.4 |
| 578 | 3 | Infectious and parasitic diseases with surgical intervention | 1.4 |
| 034 | 2 | Other diseases of the nervous system with CC | 0.9 |
| 053 | 2 | Breast and mastoid surgery, age> 17 years | 0.9 |
| 541 | 2 | Extracorporeal membrane oxygenation or tracheostomy with mechanical ventilation ≥ 96 hours or major diagnosis not related to face, mouth and neck with major surgery | 0.9 |
| 023 | 1 | Stupor state and coma of non-traumatic origin | 0.5 |
| 027 | 1 | Stupor and coma of traumatic origin, coma> 1 hour | 0.5 |
| 031 | 1 | Concussion, age> 17 years with CC | 0.5 |
| 061 | 1 | Myringotomy with tube insertion, age> 17 years | 0.5 |
| 127 | 1 | Heart failure and shock | 0.5 |
| 144 | 1 | Other diagnoses related to the circulatory system with CC | 0.5 |
| 145 | 1 | Other diagnoses related to the circulatory system without CC | 0.5 |
| 182 | 1 | Esophagitis, gastroenteritis and miscellaneous diseases of the digestive system, age >17 years with CC | 0.5 |
| 218 | 1 | Operations on lower limb and humerus except hip, foot and femur, age >17 years with CC | 0.5 |
| 265 | 1 | Skin transplant and/or debridements except for skin ulcers cellulite with CC | 0.5 |
| 385 | 1 | Infant who died or transferred to other acute care facilities | 0.5 |
| 389 | 1 | Term infants with major affections | 0.5 |
| 561 | 1 | Non-bacterial infections of the nervous system except viral meningitis | 0.5 |
| 566 | 1 | Diagnosis related to the respiratory system with assisted breathing <96 hours | 0.5 |
| 569 | 1 | Major operations on large and small intestine with CC with major gastrointestinal diagnosis | 0.5 |

Supplementary Table 4. Mean costs of acute invasive meningococcal inpatient admission by age in 2019.

| **Age group**  **(years)** | **Mean costs of admission**  **(€)** |
| --- | --- |
| 0-1 | 5,997.70 |
| 1-4 | 6,599,70 |
| 5-9 | 7,908.60 |
| 10-14 | 7,616.20 |
| 15-19 | 9,099.60 |
| 20-24 | 8,492.70 |
| 25-44 | 10,664.90 |
| 45-64 | 9,698.10 |
| ≥65 | 11,245.00 |
